# Peripheral Anterior Chamber Depth and Angle Measurements Using Pentacam After Implantation of Toric and Non-toric Implantable Collamer Lenses

**DOI:** 10.3389/fmed.2021.610590

**Published:** 2021-01-27

**Authors:** Jiao Zhao, Jing Zhao, Wen Yang, Huamao Miao, Lingling Niu, Jianmin Shang, Xiaoying Wang, Xingtao Zhou

**Affiliations:** ^1^Department of Ophthalmology, People's Hospital of Leshan, Leshan, China; ^2^Eye Institute and Department of Ophthalmology, Eye, Ear, Nose and Throat Hospital, Fudan University, Shanghai, China; ^3^National Health Center Key Laboratory of Myopia (Fudan University), Key Laboratory of Myopia, Chinese Academy of Medical Sciences, Shanghai, China; ^4^Shanghai Research Center of Ophthalmology and Optometry, Shanghai, China; ^5^Department of Ophthalmology, The Third People's Hospital of Chengdu, The Affiliated Hospital of Southwest Jiaotong University, Chengdu, China

**Keywords:** toric implantable collamer lens V4c, peripheral anterior chamber depth, anterior chamber angle, Pentacam, myopia, astigmatism

## Abstract

**Purpose:** To evaluate the characteristics of peripheral anterior chamber measurements by Pentacam after posterior implantable collamer lenses (ICL) and toric ICL (TICL) with central hole (V4c) implantation.

**Methods:** Prospective, non-randomized consecutive case series. Forty-six patients undergoing ICL implantation in one eye (Group A) and identically sized TICL in the contralateral eye (Group B) in the Refractive Surgery Center of Eye and ENT Hospital of Fudan University were prospectively included. According to ICL/TICL size, these eyes were further divided into four subgroups. Peripheral anterior chamber depth (PACD) and angle (ACA) in nasal and temporal sides were measured using Pentacam pre-operatively and 12-month post-operatively.

**Results:** The safety indices were 1.34 ± 0.32 and 1.25 ± 0.16 and the efficacy indices were 1.20 ± 0.24 and 1.19 ± 0.19 for ICL and TICL groups, respectively. There was no significant difference in pre-operative PACD or ACA between the two groups. Post-operative PACD and ACA were significantly lower than pre-operative values. Variations of PACD and ACA of TICL group were significantly larger than those of ICL group. The change of ACA for 13.2 mm lenses was significantly larger than that of 12.6 mm lenses. Pre-operative CACD and vault were significantly associated with post-operative PACD, while pre-operative ACA and vault were significantly associated with post-operative ACA.

**Conclusions:** Variations of PACD and ACA were greater in eyes after TICL (V4c) implantation compared with identically sized ICL (V4c) implantation and with larger size than smaller size lens implantation. Pre-operative anterior chamber structure and vault affect post-operative PACD and ACA.

## Introduction

Implantation of the implantable collamer lens (ICL)/ toric ICL (TICL) (V4c) with a central port is preferred over corneal refractive surgery by refractive surgeons and patients for its reversibility and excellent visual quality (1–3). ICL is positioned in the ciliary sulcus and protrudes forward to form a vault, resulting in post-operative narrowing of the anterior chamber angle (ACA) width and decreasing the central anterior chamber depth (CACD) (4).

ACA is the key anatomic parameter determining the risk for primary angle closure glaucoma (PACG) (5). Peripheral anterior synechiae and peripheral angle closure (PAC) become significant possibilities when drainage angle is ≦ grade 2 (~20°) (6, 7). In addition to ACA, peripheral anterior chamber depth (PACD) shows good sensitivity for detecting eyes at risk for angle closure (8). Extremely shallow anterior chamber depth (ACD) or narrow ACA leads to the possibility of angle closure glaucoma (ACG) and extraction of the ICL/TICL (9, 10). For these reasons, prediction and monitoring of post-operative ACA and PACD in the long term are essential to improve the safety of ICL/TICL (V4c) implantation.

Biometric studies have demonstrated that ACD significantly correlates with PAC and PACG (11–13). The Pentacam allows for quantitative measurements of the corneal topography, corneal thickness, ACA, ACD at any point, and anterior chamber volume (ACV). Pentacam has been used to screen eyes suspected of having PAC (13, 14). In addition, White-to-white (WTW) distance and CACD measured by Pentacam are crucial parameters for sizing ICL/TICL (V4c). Nevertheless, there was only one report on ACA measurement using Pentacam after ICL/TICL (V4c) implantation (15). Furthermore, there have been no comparative specialized studies on PACD or ACA after ICL and TICL (V4c) implantation.

Therefore, in the present study, we performed a prospective, non-randomized contralateral case comparison study to explore the characteristics of PACD and ACA after implantation of ICL/TICL (V4c) with various sizes. Factors that affect post-operative PACD and ACA were also analyzed.

## Patients and Methods

### Patients

This was a prospective, non-randomized consecutive case series study. Patients who underwent routine pre-operative examinations for ICL/TICL implantation and subsequent ICL (V4c) implantation in one eye (Group A) and an identically-sized TICL (V4c) implantation in the contralateral eye (Group B) between August 2018 and June 2019 were included.

TICL was selected if astigmatic diopter was beyond 0.75 D, or the percentage of astigmatic diopter to spherical diopter was higher than 10%, or the CDVA could be achieved beyond 2 lines if the astigmatic diopter was corrected by TICL. Regardless of whether TICL or ICL was selected, thorough examinations were conducted, and approval was obtained from all patients.

Groups A and B were further divided into four subgroups: A_1_ (12.6 mm ICL), A_2_ (13.2 mm ICL), B_1_ (12.6 mm TICL), and B_2_ (13.2 mm TICL), based on the size of the ICL/TICL implanted.

This study was approved by the ethics committee and followed the tenets of the Declaration of Helsinki. Written informed consent was obtained from each patient after explanation of the nature and possible consequences of the study.

### Examinations

Pre-operative routine ophthalmic examinations were performed as follows: (1) uncorrected distance visual acuity (UDVA), subjective refraction, corrected distance visual acuity (CDVA), intraocular pressure (IOP) measured using a tonometer (Canon Full Auto Tonometer TX-F; Canon, Tokyo, Japan), slit-lamp examination, axial length, fundus examination, endothelial cell density (ECD) (SP. 2000P; Topcon, Tokyo, Japan) were completed; and (2) horizontal sulcus-to-sulcus (STS) distance was measured using ultrasound biomicroscopy (UBM) (BME-300, MEDA, Tianjin, China). Eyes with ciliary or iris cysts were noted.

**Figure 1:**
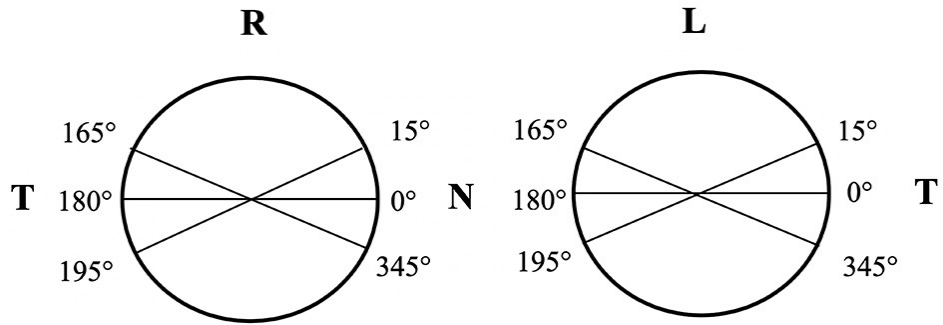
A schematic diagram for calculation of PACD/ACA in nasal and temporal sides of right and left eyes. Arithmetical mean of PACD and ACA at 0, 15, 345° meridians was considered as the value of the nasal side (NPACD/NACA) in the right eye and temporal side (TPACD/TACA) of the left eye. The arithmetical mean of PACD and ACA at 180, 165, 195° meridians was considered as TPACD/TACA of the right eye and NPACD/NACA of the left eye.

For Pentacam (OCULUS Optikgeräte GmbH, Wetzlar, Germany) examinations: PACD (defined as the ACD at 4 mm from the corneal apex) at 6 points and ACA along the 0, 15, 165, 180, 195, and 345° meridians were measured in each eye. The arithmetical means of PACD and ACA at 0, 15, and 345° meridians were considered the values of the nasal side (NPACD/NACA) in the right eye and temporal side (TPACD/TACA) of the left eye. The arithmetical means of PACD and ACA at 180, 165, and 195° meridians were considered the TPACD/TACA of the right eye and the NPACD/NACA of the left eye (Figure 1). WTW, central corneal thickness, flat keratometry (Kf), steep keratometry (Ks), CACD, pupil diameter (PD), and clear lens rise defined as the distance between the anterior pole of crystalline lens and the horizontal iris plane were also recorded.

### ICL/TICL (V4c) Implantation

ICL/TICL sizing was based primarily on WTW and CACD measurements with Pentacam HR, as recommended by the Staar surgical calculator (http://www.staarvision.com).

All surgeries were performed by two experienced surgeons using the same technique. Binocular procedures were conducted successively, and the right eye was operated on first. Surgical procedures were as previously described (16). Eyes of Group A were implanted with ICL (V4c) in the horizontal axis while eyes of Group B were implanted with TICL (V4c) with rotation of axis within 13 degrees (4.78 ± 3.02°, range: 0–13°).

### Follow-Up

The patients were followed up for 12.13 ± 4.28 months (range: 9–17 months). Follow-up examinations included assessments of UDVA, CDVA, refractive power, ECD, IOP, PACD, ACA, and vault [determined as the distance between the anterior surface of the crystalline lens and the posterior surface of ICL/TICL (V4c) on the optical axis using the Pentacam].

### Statistical Analyses

All statistical analyses were performed using SPSS 23.0 software (IBM, Armonk, NY, US). The normality of all data was first checked using the Shapiro–Wilk-test. Paired *t*-tests or Wilcoxon signed-rank-tests were used to compare pre- and post-operative data and data between different groups. Pearson's correlation or Spearman's rank correlation were performed to determine the associations between age, spherical equivalence, pre-operative CACD, PACD, ACA, PD, Kf, Ks, corneal posterior radius, astigmatism axis, axial length, WTW, horizontal STS, axis rotation of TICLs, post-operative PACD, ACA, and vault. Multiple stepwise regression analysis was performed to predict post-operative PACD/ACA using significant correlation factors in Pearson's correlation or Spearman's rank correlation analysis as independent variables. *P* < 0.05 was considered statistically significant.

## Results

Ninety-two eyes of 46 patients who underwent ICL (V4c) implantation in one eye (Group A, 46 eyes) and an identically sized TICL (V4c) implantation in the contralateral eye (Group B, 46 eyes) were included. Pre-operative demographic data and pre-operative ocular measurements are displayed in Tables 1, 2.

**Table 1:** Pre-operative patient demographic data in group A, B and subgroups.

	**Sex**		**Eye**		**Age (years)**	**Spherical (D)**	**Cylindrical (D)**	**SE (D)**
	**Male**	**Female**	**Right**	**Left**				
Group A (ICL)	18	28	26	20	25.78 ± 4.09	−8.97 ± 2.72	−0.14 ± 0.22	−9.05 ± 2.70
Group A1 (12.6 mm)	9	15	14	10	26.58 ± 4.78	−8.92 ± 2.72	−0.17 ± 0.23	−9.02 ± 2.70
Group A2 (13.2 mm)	9	13	12	10	24.91 ± 0.96	−8.64 ± 2.50	−0.09 ± 0.18	−8.70 ± 2.48
Group B (TICL)	18	28	20	26	25.78 ± 4.09	−8.51 ± 3.08^*^	−1.26 ± 0.38^*^	−9.14 ± 3.09
Group B1 (12.6 mm)	9	15	10	14	26.58 ± 4.78	−8.34 ± 3.13	−1.14 ± 0.27	−8.91 ± 3.12
Group B2 (13.2 mm)	9	13	10	12	24.91 ± 0.96	−8.35 ± 2.64	−1.39 ± 0.48	−9.05 ± 2.68

D, Diopter; SE, Spherical equivalence.

* *P < 0.05: Group A vs. Group B*.

**Table 2:** Pre-operative ocular measurements in group A, B and subgroups.

	**WTW (mm)**	**Kf (D)**	**Ks (D)**	**Rfp** **(mm)**	**Rsp** **(mm)**	**Rmp** **(mm)**	**CCT** **(μm)**	**CACD** **(mm)**	**PD** **(mm)**	**CLR** **(μm)**	**ACV** **(μl)**	**hSTS** **(mm)**	**Axial length (mm)**	**IOP (mmHg)**	**ECD (cells/mm^**2**^)**	**NPACD (mm)**	**TPACD (mm)**	**NACA (degree)**	**TACA (degree)**
Group A (ICL)	11.62 ± 0.29	43.01 ± 1.35	43.84 ± 1.27	6.55 ± 0.19	6.20 ± 0.22	6.38 ± 0.20	519.26 ± 39.27	3.25 ± 0.21	3.34 ± 0.53	360.47 ± 131.66	205.52 ± 28.88	11.78 ± 0.51	27.16 ± 1.32	15.05 ± 2.37	2710.02 ± 263.66	1.83 ± 0.31	2.19 ± 0.29	39.89 ± 5.16	43.25 ± 5.76
Group A1 (12.6 mm ICL)	11.42 ± 0.19	43.51 ± 1.26	44.17 ± 1.18	6.46 ± 0.12	6.11 ± 0.15	6.29 ± 0.11	514.29 ± 37.58	3.13 ± 0.18	3.26 ± 0.54	378.18 ± 150.63	188.58 ± 24.49	11.52 ± 0.46	26.82 ± 1.05	14.72 ± 2.33	2702.61 ± 256.46	1.66 ± 0.28	2.01 ± 0.23	40.07 ± 5.71	41.86 ± 5.99
Group A2 (13.2 mmICL)	11.84 ± 0.21^**^	42.46 ± 1.25^*^	43.48 ± 1.29	6.65 ± 0.20^*^	6.29 ± 0.25^*^	6.48 ± 0.22^*^	524.68 ± 41.22	3.37 ± 0.10^**^	3.44 ± 0.51	341.90 ± 108.93	224.00 ± 21.10^**^	12.06 ± 0.41^**^	27.53 ± 1.50	15.40 ± 2.43	2717.77 ± 276.82	2.02 ± 0.23^**^	2.38 ± 0.24^**^	39.69 ± 4.61	42.62 ± 5.13
Group B (TICL)	11.63 ± 0.28	42.83 ± 1.32^*^	44.20 ± 1.41^**^	6.55 + 0.23	6.03 ± 0.96	6.21 ± 1.01	520.63 ± 37.61	3.24 ± 0.26	3.27 ± 0.54	366.42 ± 132.16	203.48 ± 31.76	11.80 ± 0.46	27.09 ± 1.44	15.06 ± 1.96	2741.22 ± 254.19	1.86 ± 0.36	2.22 ± 0.31	40.38 ± 5.66	42.26 ± 5.55
Group B1 (12.6 mmT ICL)	11.44 ± 0.19	43.37 ± 1.25	44.58 ± 1.34	6.44 ± 0.15	6.10 ± 0.12	5.95 ± 1.36	516.67 ± 37.71	3.12 ± 0.18	3.20 ± 0.56	380.91 ± 150.52	184.46 ± 24.49	11.57 ± 0.33	26.68 ± 1.17	14.89 ± 2.03	2729.43 ± 229.97	1.66 ± 0.28	2.11 ± 0.26	40.08 ± 6.65	44.25 ± 5.59
Group B2 (13.2 mmT ICL)	11.84 ± 0.22^**^	42.24 ± 1.16^*^	43.78 ± 1.38	6.68 ± 0.22^**^	5.97 ± 1.38	6.47 ± 0.22	524.95 ± 37.90	3.36 ± 0.20^**^	3.33 ± 0.53	351.24 ± 111.43	224.23 ± 25.31^**^	12.05 ± 0.46^**^	27.54 ± 1.60	15.25 ± 1.91	2,730 ± 254.17	2.08 ± 0.32^**^	2.40 ± 0.33^**^	40.70 ± 4.49	42.17 ± 5.88

WTW, White-to-white distance; Kf, Flat keratometry; Ks, Steep keratometry; Rfp, Corneal posterior flat radius; Rsp, Corneal posterior steep radius; Rfp, Corneal posterior mean radius; CCT, Central corneal thickness; CACD, Central anterior chamber depth; PD, Pupil diameter; CLR, Clear lens rise; ACV, Anterior chamber volume; hSTS, Horizontal sulcus-to-sulcus distance; IOP, Intraocular pressure; ECD, Endothelia cell density; NPACD, Peripheral anterior chamber depth in nasal side; TPACD, Peripheral anterior chamber depth in temporal side; NACA, Anterior chamber angle in nasal side; TACA, Anterior chamber angle in temporal side.

* P < 0.05 comparison between Group A and B, Group A1 and A2, Group B1 and B2;

** *P < 0.001 comparison between Group A and B, Group A1 and A2, Group B1 and B2*.

### Safety

**Figure 2:**
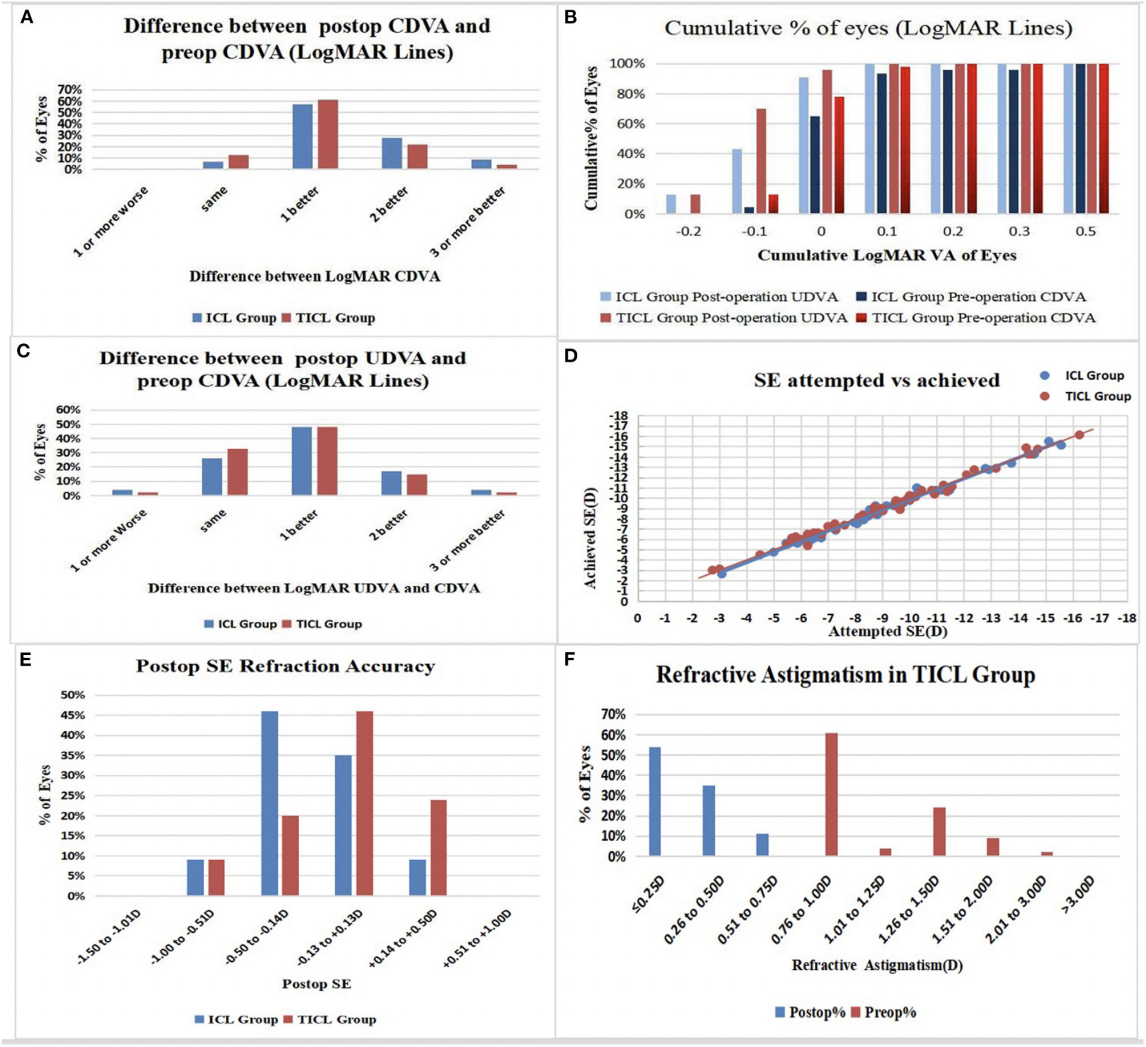
**(A)** Changes of corrected distance visual acuity (CDVA): postop vs. preop CDVA; **(B)** Cumulative percentage of the eyes attaining specified cumulative levels of uncorrected distance visual acuity (UDVA); **(C)** Changes of postop UDVA vs. preop CDVA; **(D)** Attempted spherical equivalent (SE) refraction change vs. the achieved SE refraction change at the last follow-up were plotted; **(E)** Distribution of postop SE refraction; **(F)** Distribution of preop and postop astigmatism amplitude in TICL Group.

All surgeries were uneventful, and no complications occurred during the follow-up period. The safety indices (post-operative CDVA/pre-operative CDVA) were 1.34 ± 0.32 and 1.25 ± 0.16 in ICL and TICL groups, respectively. The LogMar CDVA values at the final follow-up were −0.09 ± 0.06 in the ICL group and −0.10 ± 0.04 in the TICL group. At the final follow-up, no patient lost lines of CDVA; 63.04 and 73.91% achieved the same CDVA as pre-operatively or increased by one line; and 36.96 and 26.09% increased by two or more lines in the ICL and TICL groups, respectively (Figure 2A). Safety indices were 1.39 ± 0.32, 1.29 ± 0.17, 1.25 ± 0.17, and 1.25 ± 0.16 in the 12.6 mm ICL, 13.2 mm ICL, 12.6 mm TICL, and 13.2 mm TICL groups, respectively.

No significant differences were found for IOP or ECD between ICL and TICL groups or between subgroups at pre- and post-operative time points, as well as between pre- and post-operative time points in each group (*P* > 0.05; Tables 2, 3).

**Table 3:** Post-operative ocular measurements in group A, B, A1, A2, B1, and B2.

	**CACD-ICL (mm)**	**Vault (μm)**	**PD (mm)**	**ACV (μl)**	**IOP (mmHg)**	**ECD (cells/mm^**2**^)**	**NPACD (mm)**	**TPACD (mm)**	**ΔNPACD (mm)**	**ΔTPACD (mm)**	**NACA (degree)**	**TACA (degree)**	**ΔNACA (degree)**	**ΔTACA (degree)**
Group A (ICL)	2.45 ± 0.23	501.30 ± 161.36	3.37 ± 0.67	118.26 ± 19.60	14.60 ± 2.56	2678.37 ± 250.87	1.05 ± 0.18	1.24 ± 0.17	0.78 ± 0.25	0.94 ± 0.22	24.21 ± 4.72	26.22 ± 3.73	15.67 ± 3.49	16.00 ± 4.03
Group A1 (12.6 mm ICL)	2.39 ± 0.23	432.92 ± 182.86	3.27 ± 0.64	108.21 ± 15.78	14.31 ± 2.45	2676.78 ± 241.66	1.02 ± 0.18	1.21 ± 0.17	0.64 ± 0.17	0.79 ± 0.15	25.37 ± 5.19	27.22 ± 4.16	14.70 ± 3.68	14.64 ± 3.79
Group A2 (13.2 mmICL)	2.49 ± 0.23	575.91 ± 89.42^*^	3.47 ± 0.70	129.23 ± 17.58^**^	14.91 ± 2.70	2680.05 ± 265.85	1.08 ± 0.17	1.28 ± 0.16	0.93 ± 0.24^**^	1.09 ± 0.19^**^	22.95 ± 3.87^*^	25.12 ± 2.92^*^	16.73 ± 3.01^*^	17.49 ± 3.82^*^
Group B (TICL)	2.30 ± 0.25^**^	633.04 ± 211.11 ^**^	3.38 ± 0.60	111.43 ± 19.13^**^	14.23 ± 3.35	2707.31 ± 249.19	0.94 ± 0.17^**^	1.10 ± 0.15^**^	0.92 ± 0.31^**^	1.14 ± 0.30^**^	21.78 ± 4.22^**^	23.59 ± 3.75^**^	18.59 ± 4.01^**^	23.59 ± 3.75^**^
Group B1 (12.6 mm TICL)	2.26 ± 0.25	544.17 ± 220.76	3.31 ± 0.65	104.50 ± 16.17	14.45 ± 2.71	2704.04 ± 231.37	0.90 ± 0.17	1.10 ± 0.14	0.75 ± 0.19	1.10 ± 0.24	22.95 ± 4.89	25.06 ± 3.83	0.75 ± 0.19	1.10 ± 0.24
Group B2 (13.2 mm TICL)	2.34 ± 0.25	730.00 ± 152.47^*^	3.47 ± 0.54	119.00 ± 19.56^*^	14.01 ± 3.92	2710.73 ± 272.27	0.98 ± 0.17	1.11 ± 0.16	1.10 ± 0.32^**^	1.29 ± 0.30^*^	20.50 ± 2.95^*^	21.99 ± 2.99^*^	1.10 ± 0.32^*^	1.29 ± 0.30

CACD-ICL, Central anterior chamber depth from corneal endothelium to anterior surface of ICL/TICL; PD, Pupil diameter; ACV, Anterior chamber volume; IOP, Intraocular pressure; ECD, Endothelia cell density; NPACD, Peripheral anterior chamber depth in nasal side; TPACD, Peripheral anterior chamber depth in temporal side; NACA, Anterior chamber angle in nasal side; TACA, Anterior chamber angle in temporal side.

* P < 0.05 compared between group A and B, group A1 and A2, group B1 and B2;

** *P < 0.001 compared between group A and B, group A1 and A2, group B1 and B2*.

### Efficacy and Predictability

The efficacy indices (post-operative UDVA/pre-operative CDVA) were 1.20 ± 0.24 and 1.19 ± 0.19 in the ICL and TICL groups, respectively. The LogMar UDVA values at the final follow-up were −0.05 ± 0.07 in the ICL group and −0.07 ± 0.06 in the TICL group. At the final follow-up, all eyes had post-operative LogMAR UDVAs of 0.5 or better, and 91.30 and 95.65% achieved better than LogMAR UDVAs of 0 in the ICL and TICL groups (Figures 2B,C).

Post-operatively, 88.23% (40 eyes) of the ICL group and 85.29% (39 eyes) of the TICL group eyes achieved within ± 0.50 D of the attempted spherical equivalence (SE). All eyes were within ± 1.00 D of the attempted SE in both groups (Figures 2D,E); 95.65% (44 eyes) in the TICL group had post-operative astigmatisms of ≤ 0.50 D (Figure 2F).

### PACD and ACA

**Figure 3:**
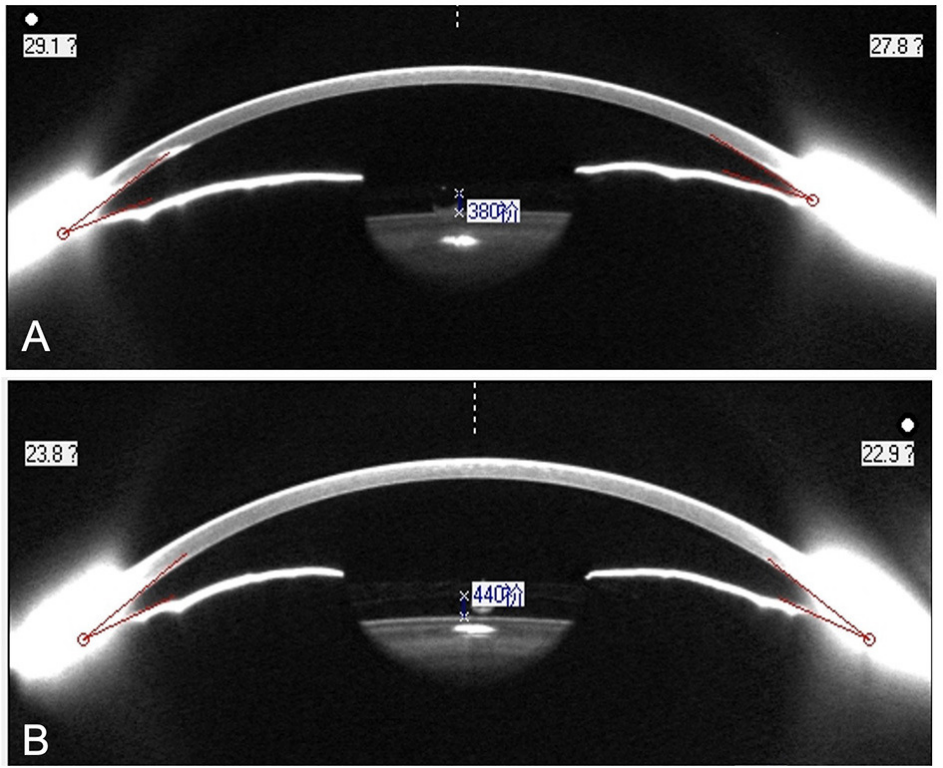
Anterior chamber image of the right eye with 13.2 mm ICL (V4c) **(A)** and left eye with 13.2 mm TICL (V4c) **(B)**. Horizontal minimal ACA was 22.2° in the right eye and 16.4° in the left eye.

After surgery, NPACD decreased by 41.91 ± 9.08% and 48.55 ± 8.76% in the ICL and TICL groups, respectively, while TPACD decreased by 42.66 ± 6.35% and 50.21 ± 7.35%, respectively. NACA decreased by 39.42 ± 7.61% and 45.97 ± 7.30%, respectively, while TACA decreased by 37.66 ± 6.82% and 44.80 ± 9.69% in the ICL and TICL groups, respectively. Variations of PACD and ACA of TICL group were significantly greater than those of ICL group. Post-operative PACD and ACA in the TICL group were significantly lower than values in the ICL group (*P* < 0.05; Table 3). One exemplary case is shown in Figure 3.

There were statistically significant differences in pre- and post-operative PACD and ACA variations between the 12.6 and 13.2 mm ICL/TICL groups (*P* < 0.05; Table 3).

Pre-operative PACD, WTW, CACD, cylindrical power of TICL, astigmatism axis, axis rotation of TICLs and post-operative vault correlated significantly with post-operative PACD (*P* < 0.05). Pre-operative ACA, PD, size of ICL/TICL, cylindrical power of TICL, astigmatism axis, axis rotation of TICLs and post-operative vault correlated significantly with post-operative ACA (*P* < 0.05; Table 4). Corneal posterior radius showed no significant correlation with post-operative PACD and ACA in univariate correlation analysis.

**Table 4:** Correlations between post-operative anterior chamber measurements and different ocular factors examined in the study group.

	**Pre-operative ACA**	**Pre-operative PACD**	**CACD**	**WTW**	**ICL/TICL Size**	**Cylindrical power**	**Astigmatism axis**	**Axis rotation of TICL**	**PD**	**Vault**
Post-NPACD	/	0.495^**^	0.444^**^	0.306^*^	/	0.235^*^	−0.233^**^	−0.238^**^	/	−0.443^**^
Post-NACA	0.638^**^	/	/	/	−0.209^*^	0.257^*^	−0.203^**^	−0.257^**^	−0.220^*^	−0.266^*^
Post-TPACD	/	0.415^**^	0.366^**^	0.264^*^	/	0.338^*^	−0.262^**^	−0.227^**^	/	−0.394^*^
Post-TACA	0.402^**^	/	/	/	−0.303^*^	0.306^*^	−0.307^**^	−0.288^**^	−0.304^*^	−0.273^*^

CACD, Central anterior chamber depth; PD, Pupil diameter; WTW, White-to-white distance; NPACD, Peripheral anterior chamber depth in nasal side; TPACD, Peripheral anterior chamber depth in temporal side; NACA, Anterior chamber angle in nasal side; TACA, Anterior chamber angle in temporal side.

* P-value of correlation coefficient < 0.05;

** *P-value of correlation coefficient < 0.001*.

Results of the multiple stepwise regression analysis are displayed in Table 5. Factors significantly associated with post-operative NPACD/TPACD included pre-operative CACD and post-operative vault (NPACD adjusted R^2^ = 0.368; TPACD adjusted R^2^ = 0.296). Factors significantly associated with post-operative NACA/TACA included pre-operative NACA/TACA, and post-operative vault (NACA adjusted R^2^ = 0.665; TACA adjusted R^2^ = 0.294).

**Table 5:** Results of multiple stepwise regression analysis for prediction of post-operative PACD and ACA.

	**Predictors**	**Unstandardized coefficients**	**Standardized coefficients**	***P*-value**
NPACD	Constant	−0.417		0.096
	Pre-operative CACD	0.482	0.626	0.000
	Vault	0.00	−0.373	0.005
	R^2^ = 0.397, Adjusted R^2^ = 0.368			
TPACD	Constant	0.139		0.608
	Pre-operative CACD	0.355	0.542	0.000
	Vault	0.00	−0.397	0.004
	R^2^ = 0.328, Adjusted R^2^ = 0.296			
NACA	Constant	6.743		0.608
	Pre-operative NACA	0.506	0.679	0.000
	Vault	−0.009	−0.427	0.000
	R^2^ = 0.680, Adjusted R^2^ = 0.665			
TACA	Constant	18.332		0.000
	Vault	−0.008	−0.471	0.001
	Pre-operative TACA	0.244	0.375	0.005
	R^2^ = 0.325, Adjusted R^2^ = 0.294			

NPACD, Peripheral anterior chamber depth in nasal side; CACD, Central anterior chamber depth; TPACD, Peripheral anterior chamber depth in temporal side; NACA, Anterior chamber angle in nasal side; TACA, Anterior chamber angle in temporal side.

*Variables in the table body are ordered according to the strength of the contribution, which was based on the standardized partial regression coefficient*.

### Iris and Ciliary Body Cysts

Iris and ciliary body cysts were found in 14 eyes of 8 patients (binocular cysts in 6 patients and monocular cysts in 2 eyes) using UBM examinations. There were nine eyes with a single cyst and five eyes with multiple cysts. All cysts were located in horizontal direction. Post-operative IOP, CACD, and vault were 13.47 ± 2.21 mmHg, 2.07 ± 0.12 mm, and 693.33 ± 126.59 μm, respectively. Pre- and post-operative NPACD values were 1.64 ± 0.38 mm and 0.77 ± 0.15 mm, pre- and post-operative NACA were 37.07 ± 4.66° and 19.13 ± 3.65°, while pre- and post-operative TPACD were 2.09 ± 0.22 mm and 1.11 ± 0.24 mm, TACA were 41.06 ± 6.56° and 23.45 ± 5.52°, respectively. NACA, TACA, NPACD, TPACD decreased by 48.64 ± 4.34%, 43.04 ± 9.40%, 52.93 ± 4.25%, and 46.67 ± 9.31%, respectively, compared with pre-operative values. None of the eyes had PAC or high IOP.

## Discussion

The present study was the first consecutive case series and contralateral eye comparison designed to investigate PACD and ACA after ICL and TICL (V4c) implantation. After 1 year of implantation, NPACD, TPACD, NACA, and TACA all decreased significantly in ICL and TICL (V4c) groups. Post-operative PACD and ACA for TICL (V4c) were significantly lower than those for ICL (V4c). Variations of these values for TICL (V4c) were significantly larger than those of ICL (V4c), although pre-operative binocular ocular measurements such as SE, WTW, and CACD remained constant. Vault for TICL (V4c) was significantly higher than that for ICL (V4c). We speculated that the higher vault might push the iris forward, resulting in more changes of anterior chamber structure after TICL (V4c) implantation. Results of correlation analysis and stepwise multivariate regression analysis validated the correlation between PACD/ACA and vault. The rotation of TICL (V4c) significantly correlated with post-operative PACD/ACA in univariate correlation analysis, however no significant correlation was found in stepwise multivariate regression analysis in the present study.

Safety of ICL/TICL implantation has been a persistent concern (17–19). PACD and ACA in the ideal range are prerequisites for the safety of post-operative surgery. Zeng et al. (9) reviewed 616 myopic eyes with the previous version of ICL/TICL (V4) implantation and found eight eyes with ICL/TICL exchange for high vault leading to shallow ACD with angle closure in any quadrant or larger PD than pre-operative measurements with severe night glare. Garcia-De la Rosa et al. (20) found significant reductions in the iridocorneal angle after ICL/TICL (V4c) implantation in mesopic, photopic, and scotopic conditions. These findings suggest that it is worth investigating changes of anterior chamber structure after ICL/TICL (V4c) implantation so as to improve surgical quality and safety.

In the present study, the safety and efficacy indices were 1.34 ± 0.32 and 1.20 ± 0.24 for ICL (V4c), and the indices were 1.25 ± 0.16 and 1.19 ± 0.19 for TICL (V4c). Previously, our team reported a post-operative safety index for ICL (V4c) of 1.80 ± 0.89 and an efficacy index of 1.54 ± 1.07 (21). The present study further demonstrated excellent safety and efficacy of variously sized ICL/TICL (V4c). At the final follow-up, 88.23 and 85.29% of eyes for ICL (V4c) and TICL (V4c), respectively, were within ± 0.50 D of the attempted SE, while all eyes from both groups were within ± 1.00 D in the present study. Garcia-De la Rosa et al. (20) reported that, among 76 eyes implanted with ICL/TICL(V4c), 54% were within ± 0.50 D for average SE and 84% were within ± 1.00 D at 12 months post-operatively. The predictability of SE values in the present study was better than that reported by Garcia-De la Rosa et al., possibly due to the discrepancy of the maximum spherical and astigmatic diopter of eyes (−14.00 DS and −2.50 DC in the present study vs. −22.25 DS and −7.00 DC in Garcia-De la Rosa et al.'s study).

ICL (V4c) was horizontally placed in the ciliary sulcus, and axis rotation of TICL (V4c) was within 13° in the present study. PACD and ACA significantly decreased after ICL/TICL (V4c) implantation according to Pentacam HR examinations. Our results were similar to those in former reports of post-operative anterior chamber change in response to different types of phakic intraocular lens (IOL) implantation. Javaloy et al. (22) reported that peripherally shallow anterior chamber appeared to be a decisive factor for a 28.78% decrease of endothelium after iris claw phakic IOL implantation during the 5-year follow-up. In another study, Benda et al. (23) reported CACD and ACA width decreased 3 years after ICL (ICH V3) implantation compared with pre-operative measurements using Pentacam. Chung et al. (24) reported a 31.8% trabecular-iris angle (TIA) reduction detected by UBM and a 41.5% angle opening distance at 500 μm from the scleral spur (AOD_500_) decreased at 1 month after ICL (V4) implantation, and no further reduction was observed thereafter in 2-year follow-up. Fernandez-Vigo et al. (25) found significant TIA narrowing of 34.5–42% and post-operative AOD_500_ decreasing of 50.3–58.4% at 3 months post-operatively and no further decrease in 2-year follow-up. Despite horizontal placement of ICL, angle narrowing was similar in both horizontal and inferior quadrants. It is speculated that angle narrowing after ICL (V4c) is caused by the pushing forward of iris in the crystal optical area due to the concave optical area on the anterior surface of ICL being larger than the pupil. In the present study, percentage of PACD decreasing and ACA narrowing were similar to AOD_500_ and TIA decreasing in V4c study by Fernandez-Vigo (25). Primary angle closure becomes a significant possibility when ACA below 20° (6). Obviously, post-operative PACD and ACA in the present study decreased within the safety range. In previous UBM and anterior segment optical coherence tomography (AS-OCT) studies, AOD_500_ decreased by a larger percentage than did TIA. In the present study, post-operative PACD decreased by a larger percentage than did ACA.

In the present study, post-operative ACA for 13.2 mm lenses was significantly lower than 12.6 mm lenses, and the change of ACA for 13.2 mm lenses was larger than that of 12.6 mm lenses, suggesting a greater effect of larger-sized lenses on ACA. To our knowledge, this is the first report to compare the changes of PACD and ACA between ICL/TICL (V4c) with different sizes (12.6 vs. 13.2 mm). Zeng et al. (9) recorded outcomes after implantation of ICL/TICL (V4) of different sizes without central hole (11.5, 12.0, and 12.5 mm) and found that eyes with larger-sized phakic interocular lenses were more likely to need ICL/TICL exchange for excessively high vault, resulting in ACA closure in any quadrant. In the present study, the mean vault of 13.2 mm lenses was significantly higher than that of 12.6 mm lenses, which was within the safe range. WTW and CACD are the most important factors in determining the ICL/TICL (V4c) size using the Staar calculator software; consequentially, larger-sized lenses would be selected for eyes with larger WTW and deeper CACD. No significant difference was found in pre-operative ACA among the different subgroups, although the values of WTW, CACD, STS, and ACV in the 13.2 mm groups were significantly larger than those of the 12.6 mm ICL/TICL (V4c) groups. These findings suggest that ACA should be taken into consideration while selecting larger-sized V4c lenses.

In this study, stepwise multiple regression analysis revealed that pre-operative CACD and post-operative vault were significantly associated with post-operative PACD. Pre-operative ACA, and post-operative vault were significantly associated with post-operative ACA. Fernandez-Vigo et al. (25) identified pre-operative TIA, age, SE, CACD, axial length, and WTW as predictors of TIA at 1-month post-ICL (V4c) implantation using Fourier-domain OCT (FD-OCT). In a subsequent 2-year follow-up report, Fernandez-Vigo (26) identified pre-operative TIA, age, sex, SE, IOL size, iris thickness at the perpendicular point 500 μm to the scleral spur, and WTW affected post-operative TIA. Lee et al. (27) reported that ICL size, STS, age, and mean K readings were determinants of post-operative vault. In addition to pre-operative ocular measurements, post-operative vault was identified as an important determinant of both post-operative PACD and ACA in the present study, respectively, indicating higher vault might push ICL/TICLs more forwardly and resulting in narrower ACA or shallower PACD. In 2010, Lindland et al. (28) first reported that the vault after toric ICL (TICL) (V4 model) implantation was higher than that of ICL (V4 model) and they presumed that addition of a cylindrical lens to the TICL optic zone may contribute to vault difference. In another study conducted by our team, we investigated the reason of ICL/TICL vault difference using multivariate parameters statistical processing and found that cylindrical power of TICL might contribute to this difference (29). We hope our conclusion will make some significance in ICL/TICL implantation surgery. Although different equipment were applied, the present study of PACD and ACA measured by Pentacam showed similar results as those of the FD-OCT study of TIA and AOD_500_ in Fernandez-Vigo's reports, suggesting that changes of anterior chamber parameters could be predicted based on pre-operative ocular measurements (25, 26).

Post-operative NPACD, TPACD, NACA, and TACA were lower than pre-operative measurements in eyes with iris and/or ciliary body cysts in the present study. Li and associates (30) found no significant differences in post-operative AOD_500_ and TIA between eyes with and without cysts. In Li's study, cysts were found predominantly in the inferior and temporal quadrants, while cysts were primarily located in the horizontal direction in the present study. Nevertheless, further research on relationships of cysts' location, size, ICL/TICL placement and PACD, and ACA change is of great importance to improve safety of ICL/TICL implantation.

The current study has some limitations. First, it utilized a relatively small sample size for few numbers of patients satisfying contralateral eye comparison design. Second, only two sizes of ICL/TICL (V4c) were analyzed, as 12.6 and 13.2 mm ICL/TICL (V4c) were selected frequently for these included patients. Future studies should be conducted with lager sample size and various lens sizes so as to make a comprehensive comparison.

In conclusion, it is possible to measure PACD and ACA using the Pentacam in ICL/TICL (V4c) implanted eyes. Pre- and post-operative variations of PACD and ACA were greater in eyes after TICL (V4c) implantation than with ICL (V4c) implantation and implantations of larger-sized lenses compared with smaller lenses. Post-operative PACD and ACA correlated with pre-operative anterior chamber structure and vault.

## Data Availability Statement

The raw data supporting the conclusions of this article will be made available by the authors, without undue reservation.

## Ethics Statement

The studies involving human participants were reviewed and approved by the institutional ethics board of Eye and ENT Hospital of Fudan University (No. 2013015-1) and informed consent was taken from all the patients after a complete description of the study. The patients/participants provided their written informed consent to participate in this study.

## Author Contributions

The study concept and design were formulated by JiaZ, JinZ, and XZ. Data collection was done by JiaZ, JinZ, WY, HM, LN, JS, XW, and XZ. Analysis and interpretation of data was undertaken by JiaZ, JinZ, and XZ. Drafting of the manuscript was carried out by JiaZ, JinZ, and XZ. Critical revision of the manuscript was done by JiaZ, JinZ, and XZ. Supervision was done by XZ. All authors contributed to the article and approved the submitted version.

## Conflict of Interest

The authors declare that the research was conducted in the absence of any commercial or financial relationships that could be construed as a potential conflict of interest.
